# COVID-19 Disease in Children: What Dentists Should Know and Do to Prevent Viral Spread. The Italian Point of View

**DOI:** 10.3390/ijerph17103642

**Published:** 2020-05-22

**Authors:** Gianmaria F. Ferrazzano, Aniello Ingenito, Tiziana Cantile

**Affiliations:** 1 Department of Neuroscience, Reproductive and Oral Sciences, School of Paediatric Dentistry, University of Naples, Federico II, Via S. Pansini, 5, 80100 Naples, Italy; ingenito@unina.it (A.I.); tizianacantile@yahoo.it (T.C.); 2 Unesco Chair on Health Education and Sustainable Development, University of Naples, Federico II, 80121 Naples, Italy; 3 Italian Society of Paediatric Dentistry (SIOI), 00199 Rome, Italy; 4 Italian Guaranteeing Authority of Childhood and Adolescence Rights–Campanian Region Section, Scientific Committee, 80121 Naples, Italy

**Keywords:** COVID-19, pediatric dentistry, children, oral health, viral spread prevention

## Abstract

Coronavirus disease 2019 (COVID-19) has spread rapidly across the globe, becoming a major public health challenge not for China only, but also for countries around the world. Despite worldwide efforts to contain viral spread, the outbreak has not been stopped yet. Among healthcare personnel, dentists seem to be at elevated risk of exposure to COVID-19. This risk is even more serious in pediatric dentistry, since affected children, frequently, present an asymptomatic, mild or moderate clinical viral infection and, therefore, they may play a major role in community-based COVID-19 transmission. To date, despite no universal guidelines are available for dental procedures in pediatric dentistry during COVID-19 outbreak, routine dental practice should be postponed and only severe dental emergencies must be treated. In the case of a dental emergency, involving a pediatric patient, dentists should be aware of which recommended management protocol can be adopted during the practice to protect patient health, to safeguard their-self and to prevent viral transmission. The aim of this paper is to provide clinical recommendations, presenting a needed tool for dentists to allow a valid and safe how-to-do protocol. Pediatric dentists should keep a high level of awareness to help patients, minimize risk and prevent viral spread.

In December 2019, a new type of coronavirus that causes pneumonia was first detected in Wuhan, China [1]. It was firstly known as 2019 novel coronavirus (2019-nCoV) [2,3,4]. On 11 February 2020, the International Committee on Taxonomy of Viruses referred to a new coronavirus capable of infecting humans as SARS-CoV-2 [5]. On the same time, the World Health Organization declared that the official name of the disease caused by this virus is COVID-19 [6].

Coronavirus disease 2019 (COVID-19) has spread rapidly across the globe, becoming a major public health challenge not for China only, but also for countries around the world [7]. In Italy, the outbreak is particularly dramatic: the first person-to-person transmission was reported on 21 February 2020, and led to an infection sequence that caused the greatest number of deaths in the world [8,9,10] until 11 April 2020, when US overtook Italy with highest coronavirus deaths. Nevertheless, Italy still remains the first country in Europe for number of deaths due to coronavirus infection.

Despite worldwide efforts to contain viral spread, the outbreak has not been stopped yet. In adults, signs and symptoms of COVID-19 may appear two to 14 days after exposure and can include: fever, cough, shortness of breath or difficulty breathing, tiredness, body aches, runny nose, sore throat. The severity of COVID-19 symptoms can range from very mild to severe. Complications can include pneumonia in both lungs, organ failure in several organs and death [11]. Literature reports on the epidemiologic characteristics and clinical features of infected children are still limited [12]. As reported by the Chinese Center for Disease Control and Prevention, most infected children appear to have a milder clinical course than infected adults [13,14,15]. Moreover, asymptomatic infections were not infrequent [12].

In a study conducted by Lu et al. children infected with SARS-CoV-2 and treated at the Wuhan Children’s Hospital were evaluated. Both symptomatic and asymptomatic children with known contact with persons having confirmed or suspected SARS-CoV-2 infection were evaluated. Nasopharyngeal or throat swabs were obtained for detection of SARS-CoV-2 RNA. Of the 1391 children assessed, a total of 171 (12.3%) were confirmed to have SARS-CoV-2 infection. Fever was present in 41.5% of the children at any time during the illness. Other common signs and symptoms included cough and pharyngeal erythema. A total of 27 patients (15.8%) did not have any symptoms of infection or radiologic features of pneumonia. A total of 12 patients had radiologic features of pneumonia but did not have any symptoms of infection [12]. A comprehensive study conducted by Dong et al. reported data on 2143 pediatric patients with COVID-19. Of them, 731 (34.1%) patients were identified as laboratory-confirmed cases and 1412 (65.9%) were suspected cases. For the severity of patients (including both confirmed and suspected cases), 94 (4.4%), 1091 (50.9%) and 831 (38.8%) patients were diagnosed as asymptomatic, mild or moderate cases, respectively, totally accounted for 94.1% of all cases [16].

The reason why COVID-19 cases in children are less severe than in adults is still confusing. Proposed explanations include children having a more active innate immune response, healthier respiratory tracts because they have not been exposed to as much cigarette smoke and air pollution as adults, and less underlying illnesses [17]. Furthermore, weaker ability to trigger an acute inflammatory response to SARS-CoV-2 might also contribute to the children’s better outcome [13].

Due to these specific features, the true rate of COVID-19 infection in children is almost certainly underestimated, since they might also have fewer chances to be tested for SARS-CoV-2, presenting, frequently, an asymptomatic, mild or moderate clinical course, similar to common cold. Therefore, the impact of children on viral spread should be highlighted, because they may play a major role in community-based viral transmission.

The possible COVID-19 transmission routes include: inhalation of airborne microorganisms that can remain suspended in the air for long periods; direct contact with blood; contact of conjunctival, nasal or oral mucosa with droplets and aerosols containing microorganisms generated from an infected individual and propelled by coughing, sneezing and talking; indirect contact with contaminated surfaces [18]. 

In addition, COVID-19 was identified in saliva of infected patients [11]. As suggest by Sabino-Silva et al. there is a minimum of three different pathways for COVID-19 to be present in saliva: from COVID-19 in the lower and upper respiratory tract, that enters the oral cavity together with the liquid droplets frequently exchanged by these organs; COVID-19 present in the blood can access the mouth via crevicular fluid; by major- and minor-salivary gland infection, with subsequent release of particles in saliva via salivary ducts [11].

Thus, healthcare workers, in daily physical contact with patients, face an elevated risk of exposure to COVID-19. Among healthcare personnel, dentists seem to be those at highest risk. It is necessary to ensure their safety, not only to protect patient health, but to safeguard their-self from the viral infection and to avoid viral transmission [19].

In this setting, dental procedures, in which a large number of droplets and aerosols, containing microorganisms from an infected individual, could be generated, are at high risk of cross infection between patients and dentists [7,11,18].

This risk is even more serious in pediatric dentistry, being the majority of COVID-19 infected patients asymptomatic or mild and moderate symptomatic [12]. To date, despite no universal guidelines are available for dental procedures during the times of any epidemic, pandemic, national or global disaster, according to guidelines for pediatric dental patients from Royal College of Surgeons of England and, more recently, from the American Academy of Pediatric Dentistry (AAPD), dental treatments have completely stopped or significantly decreased in several affected countries [20,21,22]. 

As underlined by Mallineni et al. the WHO has described a pandemic as having six different phases. Countries will be in different phases at different times; therefore, it is not possible to give universal pediatric dentistry guidelines. All the treatment choices applied to pediatric dental patients during the acute phase of COVID-19 viral spread may vary during the next phases [23].

In particular, in Italy, dental practice has been recognized as a necessary service by the Prime Minister’s decree of 22 March 2020, and its update on 25 March 2020 [24]. It has been stated that during pandemic diffusion of COVID-19, dental activities must be limited to the treatments that cannot be postponed [25].

In relation to pediatric dentistry, on 24 April 2020, the Italian Society of Pediatric Dentistry (SIOI) published on line a position paper, providing advices and recommendations for management of urgent dental care for children both during the COVID-19 pandemic and during the next phase [26].

Given that during the outbreak [7,27,28,29,30,31,32] routine dental practice should be postponed and only severe dental emergencies must be treated (such as discomfort, pain, swelling, life endangering dentigerous infection, traumatic dental injuries, etc.), in the case of a dental emergency, involving a pediatric patient, dentist should be aware of which recommended management protocol can be adopted during the practice, in order to prevent viral transmission [18,26].

First of all, dental practitioner should talk on phone with parents to obtain all the possible information both on child health status and on oral symptoms, in order to understand if dental procedure represents an urgent need and cannot be postponed (phone triage). In case of severe dental emergency, that cannot be postponed, involving a pediatric patient, once the child and one accompanying person only enter the dental office, body temperature should be measured, using infra-red thermometers. Then, they should be provided with medical masks and shoe-cover. Child and accompanying person are requested to wash hands with water and soap and to apply an alcoholic solution on hands later. Furthermore, the accompanying person should re-answer the questions on child health status [7,18,33].

Dental emergency appointments must be organized in order that no more than one pediatric patient, together with the accompanying person, should wait in the pre-operative room at a time.

Dental staff members should check their body temperatures before work. Hand washing should be implemented [7]. In particular, dental practitioners should wash their hands before child examination, before and after dental treatments. In addition, they should avoid touching their eyes, mouth and nose [18]. According to the indications of the National Association of Italian Dentists (ANDI), personal protective equipment, including eyewear, masks, gloves, caps, face shields, surgical clothes, shoe-cover, should be worn [7,18,34]. 

Validated rapid-response tests to COVID-19 infection for dental staff and dental pediatric patients before the start of any dental emergency procedures should be useful, but those currently available have a high proportion of false negatives [35]. 

Since dental procedures derived droplets and aerosols, containing microorganisms from a potential infected child, can contaminate environmental surfaces, the clinical setting should be cleaned and disinfected after every clinical session. Furthermore, other rooms should also be frequently cleaned and disinfected, including door handles, chairs, desks, screens, keyboard, phones, lamps, etc. [18,34]. During dental procedure on pediatric patients, a series of measures should be adopted.

Before the start of each dental treatment, staff members should put all the instruments and equipment required onto a tray to avoid environmental contamination during the procedure. 

For children able to split, pre-procedural mouth rinse with 0.5%–1% hydrogen peroxide should be used, as it has non specific virucidal activity against corona viruses [36]. Dentists should avoid or minimize operations that can produce droplets or aerosols: four hands technique, rubber dam, double and high-volume saliva ejectors, anti-retraction hand-pieces, hand instruments are strongly recommended in order to contrast viral spread [7,18,26]. However, in extreme situations, an extraction may be the preferred treatment option for children with pulpal symptoms (in deciduous dentition) to reduce the need for aerosol generating procedures [21].

According to the recommendations from the Royal College of Surgeons of England, dental trauma in permanent dentition, like avulsion, severe luxation, crown-root fracture, complicated crown fracture with pulp involvement, should be managed as soon as possible. For avulsed teeth, storage medium, extra-oral time, degree of tooth maturity, patient co-operation should be considered prior to replantation. For severely luxated teeth, repositioning and splinting should be the best treatment. Dental injuries in deciduous dentition needing a fast resolution are pulp exposure and severe luxation, when tooth mobility constitutes a potential airway risk and/or tooth is severely interfering with occlusion and function [21].

In case of severe dental emergencies, for those children who will not accept treatment in the chair, dental treatment performed in public hospitals under sedation and/or general anesthesia (GA) could be the best therapeutic choice to provide a safe and effective dental treatment. While waiting for dental treatment under sedation and/or GA, appropriate antibiotic should be only prescribed when needed, and, when needed, the right antibiotic should be selected and prescribed at the right dose and for the right duration, in accordance with evidence-based national and local clinical practice guidelines, to minimize the risk of developing resistance to current antibiotic regimens [37,38].

Furthermore, pediatric dentists should safeguard, above all, children with compromised systemic health, being at greater risk of complications arising from any dental infection and special needs children (for example, autistic pediatric patients), whose behavior may become impossible to manage in case of severe dental pain [21].

Additionally, according to the evolving situation, in the next future, until COVID-19 will be completely eliminated, as advised by the Italian Society of Pediatric Dentistry (SIOI), minimally invasive dentistry, such as atraumatic restorative treatment, sealing in carious lesions using fissure sealants, silver diamine fluoride, selective caries removal and the Hall Technique, should be taken in to consideration [23,26].

## Conclusions

In the scientific literature, there is still limited information on the model that dentists should follow for the management of emergency dental procedure on children during COVID-19 outbreak.

COVID-19 outbreak will probably change daily routine dental practice all over the world. Furthermore, at the moment, there is no evidence that personal protective equipment and dental office disinfection procedures, commonly used in dentistry until now, could be safe enough.

Actually, dental practice could need to be rethought and reorganized in order to ensure higher safety standards for both dentists and patients.

Finally, since the COVID-19 situation continues to evolve day by day, pediatric dentists should keep a high level of awareness to help patients, minimizing risk and preventing viral spread.
